# Supplementary material for: A simple model for learning in volatile environments
Source: PLoS Comput Biol. 2020 Jul 1;16(7):e1007963. doi: 10.1371/journal.pcbi.1007963 (PMC7329063; doi:10.1371/journal.pcbi.1007963)
Supplement: S1 Text — (PDF) [file pcbi.1007963.s001.pdf]

Control analyses for comparison between VKF and HGF

We first verified that although the two generative models are parameterized differently, their parameters models were chosen in comparable regimes with respect to the ultimate inference problem of tracking the latent state x_t. In particular, the median trial-by-trial change in that lower-level signal, which is defined similarly in both models based on a Gaussian random walk, was comparable. The average of this measure across all simulations for HGF and VKF was 1.84 and 1.93, respectively.

Performance of the models might depend on the parameters determining variability of the volatility signal. In the HGF and VKF, this depends on ν and λ, respectively. Therefore, we performed a second analysis in a different parameter regime by reducing ν from 0.5 (the original analysis) to 0.25 for the HGF, and by reducing λ from 0.15 (original analysis) to 0.1 for the VKF. The relative error of the HGF and VKF was 8.20% (SE=0.6%) and 3.2% (SE=0.3%), respectively. The measure of trial-by-trial changes in the lower-level signal, as defined above, for the HGF and VKF was 0.68 and 1.01, respectively, indicating that the parameters generate comparable signals, and if anything, the tracking problem for VKF is a bit harder in that the latent variable is a bit less stable.

Next, we considered the possibility that differences between the models' relative performance scores might arise not due to their particular inferential approximations, but instead due to the particle filtering algorithm used as a baseline. In particular, while it is important to use such a baseline, so as to measure performance relative to a measure of what is optimally achievable in a particular generative family, actual optimal inference is intractable, so we rely on the RBPF as a proxy. However, that algorithm is also approximate (though computationally intensive and with good convergence properties), and in principle its error might also differ between the generative processes. Accordingly, we also compared the two models relative to a second baseline independent of (and algorithmically simpler than) the RBPF, this one an idealistic baseline provided by an ideal Kalman filter augmented with an (unrealistic) oracle providing the true volatility state at each step. This construction exploits the fact that both VKF and HGF reduce to the Kalman filter given the volatility. In particular, for every timeseries generated in the original analysis, we simulated the Kalman filter in which the volatility parameter on every trial was given by the true volatility. Note that this is not a realistic inference model, as it has access to information (i.e. true volatility) that other inference models do not. This idealistic Kalman filter model was then used as the baseline to obtain a measure of relative error for both the VKF and HGF, similar to the previous analysis. Across all simulations, the relative error for the VKF and HGF was 5.5% (SE=0.4%) and 46.6% (SE=6.7%), respectively, indicating that the inference by the VKF was closer to the idealistic Kalman filter.

All these results comparing accuracy of VKF and HGF in inference on timeseries generated by their own generative models for both sets of parameters per model are summarized in the following table.

		Number of conflicts	Relative error– RBPF	Relative error– ideal Kalman	Correlation coefficient state	Correlation coefficient volatility
Set 1	HGF	82	21.4%	46.6%	1.00	0.88
	VKF	NA	2.7%	5.5%	1.00	0.95
Set 2	HGF	15	8.2%	25.8%	1.00	0.88
	VKF	NA	3.2%	5.0%	1.00	0.85

Subject-level fitting of models to empirical data

In addition to the HBI hierarchical fitting approach discussed in the main text, to verify the generality of the result, we also fit empirical data to the models at the individual per-subject level using a Laplace approximation. For the four models reported in the main text, this is equivalent to the first iteration of the HBI. We also included the particle filter (PF) model in this analysis using a simple sampling procedure (1000 samples) to first draw generative parameters of the VKF (i.e. v_0 and λ), fit the PF (10000 particles) given those parameters to obtain trial-by-trial predictions, and then fitted the parameters of the response model using the Laplace approximation. To quantify model evidence at the subject-level, we used Bayesian information criteria (BIC) to account for the generative parameters. The following table shows the results for both experiments in terms of model frequency (MF) and protected exceedance probability (PXP).

Model	Experiment 1		Experiment 2	
	PXP	MF	PXP	MF
VKF	0.85	0.40	1	0.76
HGF	0.01	0.12	0	0.05
RW	0.09	0.24	0	0.04
KF	0.04	0.21	0	0.15
PF	0.01	0.02	0	0.01
