# Supplementary material for: A simple model for learning in volatile environments
Source: PLoS Comput Biol. 2020 Jul 1;16(7):e1007963. doi: 10.1371/journal.pcbi.1007963 (PMC7329063; doi:10.1371/journal.pcbi.1007963)
Supplement: S1 Appendix — (PDF) [file pcbi.1007963.s002.pdf]

A simple model for learning in volatile environments

Payam Piray* and Nathaniel D. Daw
*ppiray@princeton.edu

1 State Space Models

Here, we give a formal treatment of the volatile Kalman filter (VKF). We begin by describing state space models, in which a sequence of observations, $\{u_i\}$ where $i = 1, ..., t$, is modeled by specifying a probabilistic relation between the observations and a sequence of latent states, $\{s_i\}$ and a Markov structure linking the latent states. A state space model assumes that i) u_t is independent of all other observations and states given s_t, and ii) s_t is independent of all other states given s_{t-1}. Thus, the joint distribution for the state space model is given by

$$p(s_1, ..., s_t, u_1, ..., u_t) = p(s_1) \prod_{i=2}^{t} p(s_i|s_{i-1})p(u_i|s_i). \tag{1.1}$$

The inference goal in the state space model is to obtain the posterior over s_t given observations. It can be shown then that regardless of the form of transition probabilities and the probabilistic relation between s_t and u_t, the posterior can be obtained recursively. First, the current state estimate is projected ahead in time. In other words, the posterior of s_t given all observations prior to t is calculated based on the previous posterior $q(s_{t-1}) = p(s_{t-1}|u_1, ...u_{t-1})$:

$$\begin{aligned} \hat{q}(s_t) = p(s_t|u_1, ..., u_{t-1}) &= \int q(s_{t-1})p(s_t|s_{t-1})\,\mathrm{d}s_{t-1} \quad \text{for } t > 1 \\ \hat{q}(s_1) &= p(s_1). \end{aligned} \tag{1.2}$$

Next, the projected estimate is adjusted by actual measurement, giving rise to

the posterior given all observations:

$$q(s_t) = \frac{1}{c_t} p(u_t|s_t)\hat{q}(s_t), \tag{1.3}$$

where $c_t = p(u_t|u_1, ..., u_{t-1})$ is constant with respect to s_t. Note that since c_t is independent of s_t, it is only needed to be calculated if one is interested in the likelihood of data, which is easy to see to be given by

$$p(u_1, ..., u_t) = \prod_{i=1}^{t} c_i. \tag{1.4}$$

Furthermore, the posterior over two consecutive states s_{t-1} and s_t is given by

$$q(s_{t-1}, s_t) = p(s_{t-1}, s_t|u_1, ..., u_t) = Cq(s_{t-1})p(s_t|s_{t-1})p(u_t|s_t), \tag{1.5}$$

where C is a normalization constant independent of s_{t-1} and s_t.

As noted above, equations (1.2-1.5) are valid for any model with conditional dependencies as given in equation (1.1) regardless of the form of $p(s_t|s_{t-1})$ and $p(u_t|s_t)$. However, the form of $p(s_t|s_{t-1})$ and $p(u_t|s_t)$ determine whether the integral in equation (1.2) is tractable and whether the process can be summarized in an efficient and simple algorithm by ensuring that $q(s_t)$ has the same functional form as $q(s_{t-1})$. Kalman filter [1] provides a tractable solution for a state space model in which transition probabilities are Gaussian with a fixed variance, which is suitable for prediction in stable environments. We discuss Kalman filter in section 2. In section 3, we build another state space model in which observations are Gaussian with a stochastic variance determined by the states [2, 3]. Furthermore, while the source of stochasticity in state transitions of Kalman filter is additive noise, the source of stochasticity in the second state space model is a multiplicative noise, which makes it appealing as a model of variance. Then, in section 4, we combine the two models to address prediction problems in volatile environments.

2 Kalman filter

Consider a state space model in which, on trial n, hidden state of the environment, x_t, is equal to its state on trial $n-1$ plus some Gaussian noise.

$$x_t = x_{t-1} + e_t, \tag{2.1}$$

where e_t is a zero-mean Gaussian random variable with variance v. Therefore, the generative probabilistic model of x_t is

$$p(x_t|x_{t-1}) = \mathcal{N}(x_t|x_{t-1}, v). \tag{2.2}$$

The initial state is also assumed to be Gaussian

$$p(x_1) = \mathcal{N}(x_1|0, v). \tag{2.3}$$

Observations are given by a Gaussian distribution:

$$p(o_t|x_t) = \mathcal{N}(o_t|x_t, \sigma^2), \tag{2.4}$$

where σ^2 is the observation variance. Using equations (1.2-1.3), we obtain:

$$q(x_t) = \mathcal{N}(x_t|m_t, w_t), \tag{2.5}$$

where

$$m_t = m_{t-1} + k_t(o_t - m_{t-1}), \tag{2.6}$$

$$w_t = (1 - k_t)(w_{t-1} + v), \tag{2.7}$$

and k_t is the learning rate (also called Kalman gain) and is given by:

$$k_t = \frac{w_{t-1} + v}{w_{t-1} + v + \sigma^2}. \tag{2.8}$$

Furthermore, we can make use of (1.5) to obtain the covariance between consecutive states, x_{t-1} and x_t:

$$w_{t-1,t} = (1 - k_t)w_{t-1}. \tag{2.9}$$

3 A tractable filter for variance

Let's now consider another state space model in which hidden state on trial t, z_t, depends on its value on previous trial, z_{t-1} multiplied by some (independent) noise, ϵ_t. Then, a Gaussian random variable, x_t, is drawn with a known mean, μ_t, and a precision given by z_t,

$$p(x_t|z_t) = h_t(z_t) = \mathcal{N}(x_t|\mu_t, z_t^{-1}). \tag{3.1}$$

Therefore, conditional dependencies indicate that equations (1.2-1.3) hold for the posterior over z_t.

Our goal in this section is to build a rich model of dynamic (inverse) variance with tractable and simple update equations. First, we inspect the functional form of $h_t(z_t)$ with respect to z_t:

$$h_t(z_t) \propto z_t^{\frac{1}{2}} e^{-\frac{1}{2}z_t(x_t-\mu_t)^2}. \tag{3.2}$$

Therefore, $h_t(z_t)$ takes a form of gamma distribution in which information about new observations is always coming through the rate and the shape is constant. Moreover, equation (1.3) indicates that if $\hat{q}(z_t)$ is a gamma distribution, then $q(z_t)$ takes the form of gamma distribution. Thus, for the moment, we assume that $q(z_t)$ is a gamma distribution with a constant shape a and a dynamic rate b_t:

$$q(z_t) = \mathcal{G}(z_t|a, b_t).$$

In the previous section, we saw that the transition noise was additive. For variance, it makes sense to assume a multiplicative noise. Thus, we suppose that the value of z_t is equal to z_{t-1} multiplied by some noise

$$z_t = z_{t-1}\epsilon_t.$$

Assuming that the noise is bounded between zero and $R \geq 1$, we can write this equation as

$$z_t = Rz_{t-1}\varepsilon_t, \tag{3.3}$$

where $\epsilon_t = R\varepsilon_t$, in which ε_t is constrained in the unit range and has a beta distribution. Assuming that conditional expectation of z_t is z_{t-1}, we obtain

$\mathbb{E}[\varepsilon_t] = R^{-1}$. A general beta distribution with this property is given by:

$$p(\varepsilon_t) = \mathcal{B}(\varepsilon_t | \eta\nu, (1-\eta)\nu), \tag{3.4}$$

where $\eta = R^{-1}$ and ν is a constant positive parameter. We show below that there is a specific value for ν, which makes the inference tractable.

Using transform theorem for random variables, the conditional distribution of z_t is given by

$$p(z_t | z_{t-1}) = \mathcal{B}(\eta z_t / z_{t-1} | \eta\nu, (1-\eta)\nu)\eta z_{t-1}^{-1} \qquad 0 < z_t < \eta^{-1} z_{t-1}.$$

Applying equation (1.2) to $\hat{q}(z_t)$, we obtain

$$\hat{q}(z_t) \propto \int z_{t-1}^{a-1} e^{-b_{t-1} z_{t-1}} (\frac{\eta z_t}{z_{t-1}})^{\eta\nu - 1} (1 - \frac{\eta z_t}{z_{t-1}})^{(1-\eta)\nu - 1} \frac{1}{z_{t-1}} \, \mathrm{d}z_{t-1}\,.$$

If we take a to be equal to ν, the integral is tractable resulting in a gamma distribution for $\hat{q}(z_t)$. Note that evaluating those terms that are independent of z_t is not even necessary because $\hat{q}(z_t)$ is a probability distribution. Therefore, we have

$$\hat{q}(z_t) = \mathcal{G}(z_t | \eta\nu, \eta b_{t-1}). \tag{3.5}$$

Combining $\hat{q}(z_t)$ with $h(z_t)$, we obtain $q(z_t)$:

$$q(z_t) = \mathcal{G}(z_t | \eta\nu + \frac{1}{2}, b_t),$$

where

$$b_t = \eta b_{t-1} + \frac{1}{2}(x_t - \mu_t)^2. \tag{3.6}$$

If we now take the shape of $q(z_t)$ to be the same as the shape of $q(z_{t-1})$, the inference is similarly tractable for all next trials. Consequently, ν is given by

$$\nu = \frac{1}{2(1-\eta)}. \tag{3.7}$$

Therefore, the posterior distribution is a gamma distribution with a fixed shape and an evolving rate

$$q(z_t) = \mathcal{G}(z_t | \nu, b_t). \tag{3.8}$$

We can also write b_t in terms of $v_t = \mathbb{E}[z_t]^{-1}$:

$$b_t = 2(1-\eta)\mathbb{E}[z_t]^{-1}.$$

Now we can also write (3.6) in terms of $v_t = \mathbb{E}[z_t]^{-1}$:

$$v_t = \eta v_{t-1} + (1-\eta)(x_t - \mu_t)^2.$$

If we define $\lambda = 1-\eta$, we can write this equation in the form of an error-correcting update rule:

$$v_t = v_{t-1} + \lambda((x_t - \mu_t)^2 - v_{t-1}). \tag{3.9}$$

It is also important to note that $\mathbb{E}[z_t]^{-1}$ under $\hat{q}(z_t)$ is given by v_{t-1} according to equation (3.5). Furthermore, as equation (1.2) indicates $\hat{q}(z_1) = p(z_1)$, we take the probability of initial state to be given by

$$p(z_1) = \mathcal{G}(z_1|\eta\nu, \eta b_0), \tag{3.10}$$

where $b_0 > 0$ is a free parameter, which we can equivalently write it according to the initial volatility value v_0. Note that as a consequence of making the inference tractable, the form of multiplicative transition noise is restricted according to equation (3.7). However, this is not a major restriction, as the mean of noise is still free to be chosen. Note that it is also possible to assume that λ decays over time, which makes the generative process more stable.

4 Volatile Kalman filter

In this section, we provide a solution to the general prediction problem in volatile environments. Specifically, consider a problem in which on trial t a latent random variable, x_t, is given by its previous value, x_{t-1}, plus some Gaussian noise with a precision given by another dynamic random variable, z_t:

$$x_t = x_{t-1} + e_t, \tag{4.1}$$

$$p(e_t) = \mathcal{N}(e_t|0, z_t^{-1}). \tag{4.2}$$

The dynamic of z_t is given by equations (3.3-3.4). The observation on trial t, o_t, is then drawn based on the value of x_t according to a normal distribution:

$$p(o_t|x_t) = \mathcal{N}(o_t|x_t, \sigma^2). \tag{4.3}$$

Therefore, the graphical model corresponding to this problem consists of two coupled chains of latent variables, x_t and z_t. Exact inference for this problem is not possible due to coupling between the two chains. However, it is possible to derive an accurate and efficient approximation by exploiting the fact that inference within each chain is tractable. Here, we employ structured variational inference techniques [4, 5] by assuming factorization between the two chains, while preserving all dependencies within each chain. Here, we assume

$$p(x_1, ..., x_t, z_1, ..., z_t|o_1, ..., o_t) \simeq q(x_1, ..., x_t)q(z_1, ..., z_t). \tag{4.4}$$

Using variational calculations, we obtain

$$\begin{aligned} q(x_1, ..., x_t) &\propto \prod_{i=1}^{t} \exp\Big(\mathbb{E}[\log p(z_i|z_{i-1})p(x_i|x_{i-1}, z_i)p(o_i|x_i)]\Big) \\ &= C \prod_{i=1}^{t} f_i(x_{i-1}, x_i) g_i(x_i), \end{aligned} \tag{4.5}$$

where all terms independent of $x_1, ..., x_t$ are absorbed into C and we have:

$$\begin{aligned} f_i(x_{i-1}, x_i) &\triangleq \exp\big(\mathbb{E}[\log p(z_i|z_{i-1})p(x_i|x_{i-1}, z_i)]\big) \\ &\propto \mathcal{N}(x_i|x_{i-1}, \mathbb{E}[z_i]^{-1}) \\ &= \mathcal{N}(x_i|x_{i-1}, v_i), \end{aligned} \tag{4.6}$$

and

$$g_i(x_i) \triangleq p(o_i|x_i). \tag{4.7}$$

Since the conditional dependencies for x_{i-1} and x_i in (4.5) is the same as those in (1.1), exact inference for $q(x_t)$ follows (1.2-1.3). Moreover, since dependencies are in Gaussian forms, we obtain $q(x_t) = \mathcal{N}(x_t|m_t, w_t)$, we can use Kalman equations (2.5-2.8) to update m_t and w_t in which we replace v in those equations with v_t.

Next, we use variational techniques to obtain $q(z_t)$:

$$\begin{aligned} q(z_1, ..., z_t) &\propto \prod_{i=1}^{t} \exp\Big(\mathbb{E}[\log p(z_i|z_{i-1})p(x_i|x_{i-1}, z_i)p(o_i|x_i)]\Big) \\ &= C\prod_{i=1}^{t} p(z_i|z_{i-1})h_i(z_i), \end{aligned} \tag{4.8}$$

where all terms independent of $z_1, ..., z_t$ are absorbed into C and

$$\begin{aligned} h_i(z_i) &\triangleq \exp\Big(\log \mathbb{E}[p(x_i|x_{i-1}, z_i)\Big) \\ &= \frac{1}{\sqrt{2\pi}} z_i^{\frac{1}{2}} \exp(-\frac{1}{2} z_i \mathbb{E}\big[(x_i - x_{i-1})^2\big]). \end{aligned} \tag{4.9}$$

Since the conditional dependencies for z_{i-1} and z_i in (4.8) is the same as those in (1.1), inference for $q(z_t)$ follows (1.2-1.3). Moreover, since dependencies between z_{i-1} and z_i have the same functional form as (3.2) and (3.3), the posterior $q(z_t)$ takes the form of Gamma distribution, in which its mean, v_t, gets updated according to $\mathbb{E}\big[(x_t - x_{t-1})^2\big]$:

$$v_t = v_{t-1} + \lambda(\mathbb{E}\big[(x_t - x_{t-1})^2\big] - v_{t-1}). \tag{4.10}$$

Since the expectation in (4.10) should be taken under the posterior $q(x_{t-1}, x_t)$, we have

$$\begin{aligned} \mathbb{E}\big[(x_t - x_{t-1})^2\big] =& \mathbb{E}\big[x_{t-1}^2\big] + \mathbb{E}\big[x_t^2\big] - 2\mathbb{E}\big[x_{t-1}x_t\big] \\ =& m_{t-1}^2 + w_{t-1} + m_t^2 + w_t - 2(m_{t-1}m_t + w_{t-1,t}) \\ =& (m_t - m_{t-1})^2 + w_{t-1} + w_t - 2w_{t-1,t}. \end{aligned} \tag{4.11}$$

These results give rise to an efficient and simple algorithm for learning in volatile environments. On every trial t, we first compute $\mathbb{E}[z_t]^{-1}$ under $\hat{q}(z_t)$, which is given by v_{t-1}. Next, we update m_t and w_t and v_t, according to equations (2.6-2.9) by replacing v with $\mathbb{E}[z_t]^{-1} = v_{t-1}$. Finally, making use of equations (4.11) and (4.10), we update volatility. Note that here, unlike typical algorithms obtained based on variational Bayes, we only update statistics once to have an efficient algorithm for learning.

5 Binary VKF

In this section, we give a formal treatment of the binary VKF, in which observations are drawn based on a Bernoulli distribution:

$$p(o_t|x_t) = \sigma(x_t)^{o_t}(1 - \sigma(x_t))^{1-o_t}. \tag{5.1}$$

where $\sigma(.)$ is the sigmoid function:

$$\sigma(x_t) = \frac{1}{1 + \exp(-x_t)}. \tag{5.2}$$

Note that for binary observations, (1.3) indicates that $q(x_t)$ is not normally distributed even if we assume that $\hat{q}(x_t)$ is normal. Therefore, some approximate strategies are required for binary observations.

A well-known method approximates $q(x_t)$ by matching its moments to those of $p(o_t|x_t)\hat{q}(x_t)$ [6, 7], which has particularly been successful for inference models of binary data [8]. For a Gaussian distribution, this means that first and second order moments should be matched. However, since matching the second order moment might lead to negative values for the variance, we assume that the variance is constant (similar to Kalman filter) and match only the mean of distributions. Formally, we take the "message" from o_t to x_t to be an unnormalized Gaussian, $g_t(x_t) \propto \mathcal{N}(x_t|\tilde{m}_t, \omega)$, in which the variance ω is a constant. We obtain $\tilde{m}_t$ by matching the mean of $q(x_t) = \mathcal{N}(x_t|m_t, w_t)$ with that of $p(o_t|x_t)\hat{q}(x_t)$, in which $\hat{q}(x_t) = p(x_t|o_1, ..., o_{t-1}) = \mathcal{N}(x_t|m_{t-1}, w_{t-1} + v_{t-1})$. We used matching techniques for Gaussian densities [6, 9] and a simple approximation for the arising integral, which is inspired by Mackay [10]:

$$m_t \simeq m_{t-1} + \sqrt{w_{t-1} + v_{t-1}}(o_t - \sigma(m_{t-1})). \tag{5.3}$$

It is also possible to approximate m_t using a slightly more complicated equation given by MacKay [10]. Using simulation analyses, however, we found that (5.3) results in a more accurate approximation. The variance of $q(x_t)$ is then given by

$$w_t^{-1} = \hat{w}_t^{-1} + \omega^{-1}, \tag{5.4}$$

which results in the binary VKF algorithm presented in the main text.

References

1. Kalman, R. E. A New Approach to Linear Filtering and Prediction Problems. *Transactions of the ASME–Journal of Basic Engineering* **82,** 35–45 (1960).
2. Gamerman, D., dos Santos, T. R. & Franco, G. C. A Non-Gaussian Family of State-Space Models with Exact Marginal Likelihood. *Journal of Time Series Analysis* **34,** 625–645 (2013).
3. West, M. On Scale Mixtures of Normal Distributions. *Biometrika* **74,** 646–648 (1987).
4. Saul, L. & Jordan, M. I. *Exploiting Tractable Substructures in Intractable Networks* in *Advances in Neural Information Processing Systems 8* (MIT Press, 1995), 486–492.
5. Ghahramani, Z & Jordan, M. I. Factorial Hidden Markov Models | SpringerLink. *Machine Learning* **29,** 245–273 (1997).
6. Minka, T. P. *Expectation Propagation for Approximate Bayesian Inference* in *Proceedings of the Seventeenth Conference on Uncertainty in Artificial Intelligence* (Morgan Kaufmann, Seattle, Washington, 2001), 362–369.
7. Boyen, X. & Koller, D. *Tractable Inference for Complex Stochastic Processes* in *Proceedings of the Fourteenth Conference on Uncertainty in Artificial Intelligence* (Morgan Kaufmann, Madison, Wisconsin, 1998), 33–42.
8. Kuss, M. & Rasmussen, C. E. Assessing Approximate Inference for Binary Gaussian Process Classification. *Journal of Machine Learning Research* **6,** 1679–1704. ISSN: ISSN 1533-7928 (Oct 2005).
9. Thomas, M. *EP: A Quick Reference* (2008). `https://tminka.github.io/papers/ep/minka-ep-quickref.pdf` (2020).
10. MacKay, D. J. C. The Evidence Framework Applied to Classification Networks. *Neural Computation* **4,** 720–736 (1992).
