# Supplementary material for: A simple model for learning in volatile environments
Source: PLoS Comput Biol. 2020 Jul 1;16(7):e1007963. doi: 10.1371/journal.pcbi.1007963 (PMC7329063; doi:10.1371/journal.pcbi.1007963)
Supplement: S2 Appendix — (PDF) [file pcbi.1007963.s003.pdf]

Although in the main text, we examined learning rates for binary outcomes in the space of the latent variable where learning occurs, it is also possible, as pointed out by a reviewer, to impute the learning rate implied by each such update in observation space. Here, we consider this alternative metric of learning rate in the observation space, α_t^o, which is defined as below:

$$\alpha_t^o = \frac{s(m_t) - s(m_{t-1})}{\delta_t}, \tag{1}$$

where m_{t-1} and m_t are the old and new mean prediction of the (latent) state variable, $s(m) = (1+\exp(-m))^{-1}$ is the sigmoid function, and δ_t is the prediction error:

$$\delta_t = o_t - s(m_{t-1}). \tag{2}$$

S1 Fig. shows that unlike the HGFs learning rate in the latent space, this quantity, α_t^o, increases during blocks of higher volatility in the probabilistic learning task with binary observations, seemingly reflecting the theoretically expected relationship between volatility and learning rate. However, this behavior actually does not arise from the models top-down inferences about volatility, but instead is directly contaminated by differences in prediction errors between the blocks. As shown in the figure, it is present even when the models volatility estimate is held fixed.

Here, we analyze the source of this contamination, demonstrating that α_t^o is contaminated by the absolute value of prediction error and therefore increases following switches regardless of volatility (and even if volatility is fixed). Both the binary VKF and binary HGF update their estimation according to the following equation:

$$m_t = m_{t-1} + \alpha_t \delta_t, \tag{3}$$

where α_t is the learning rate as defined in the main text (and plotted in Fig 6). Note that α_t is different for the VKF and the HGF, but the update rule of m_t is the same as this equation for both models. Therefore, α_t^o is given by:

$$\alpha_t^o = \frac{s(m_{t-1} + \alpha_t \delta_t) - s(m_{t-1})}{\delta_t}. \tag{4}$$

The problem arises because the numerator of α_t^o depends on the second order effects of δ_t. To see this, we expand $s(m_{t-1} + \alpha_t \delta_t)$ using Taylor series around

m_{t-1}:

$$s(m_{t-1} + \alpha_t \delta_t) = s(m_{t-1}) + \alpha_t \delta_t s'(m_{t-1}) + (\alpha_t \delta_t)^2 s''(m_{t-1}) + O(\delta_t^2) \quad (5)$$

where $O(\delta_t^2)$ contains terms higher than second order, and s' and s'' are the first and second derivatives of the sigmoid function, respectively. Substituting this equation into the right-hand side of equation (4), α_t^o becomes:

$$\alpha_t^o = \alpha_t s'(m_{t-1}) + \alpha_t^2 \delta_t s''(m_{t-1}) + O(\delta_t). \quad (6)$$

The first and second derivatives of sigmoid are given by:

$$s'(m) = s(m)(1 - s(m)), \quad (7)$$

and

$$s''(m) = s'(m)(1 - 2s(m)). \quad (8)$$

It can be easily seen that $s'(m)$ is positive regardless of m, but the sign of $s''(m)$ depends on the last term on the right hand side of (8). Therefore, by substituting s'' in equation (6), we see that the sign of second term in (6) depends on $(1-2s(m_{t-1}))\delta_t$, which is positive and large following switches in probabilistic tasks (e.g. Fig 6). In particular, there are two types of switches:

- $o_t = 1$ and m_{t-1} is negative and relatively large, then both $1 - 2s(m_{t-1})$ and δ_t are positive and large.
- $o_t = 0$ and m_{t-1} is positive and relatively large, then both $1 - 2s(m_{t-1})$ and δ_t are negative and large.

Therefore, α_t^o is influenced by the absolute value of prediction errors, $|\delta_t|$, following switches, regardless of volatility and even if volatility or α_t is fixed (S1 Fig).
