# Supplementary figures and images for: A simple model for learning in volatile environments
Source: PLoS Comput Biol. 2020 Jul 1;16(7):e1007963. doi: 10.1371/journal.pcbi.1007963 (PMC7329063; doi:10.1371/journal.pcbi.1007963)

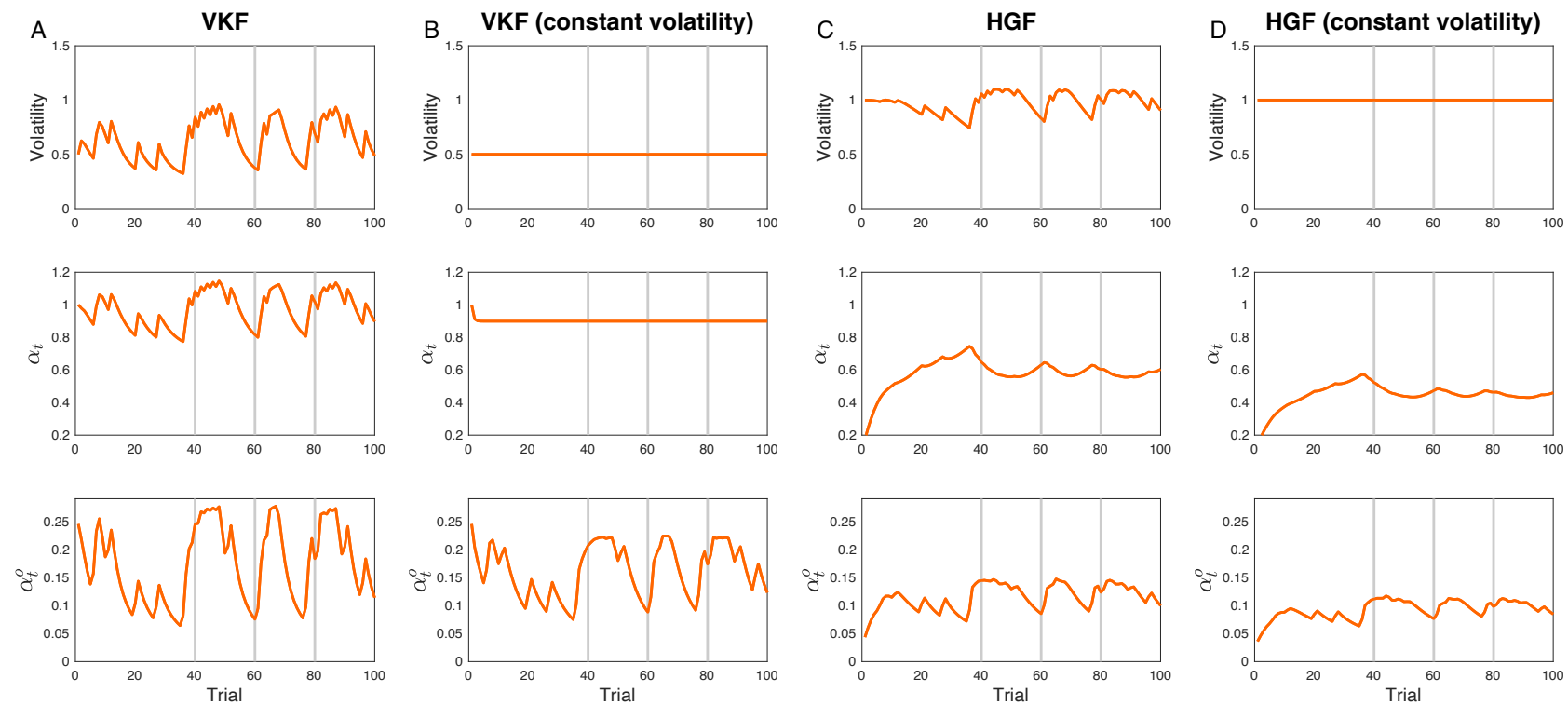

Supplement: S1 Fig — Volatility signal and learning rates are plotted for the A) VKF; B) VKF with constant volatility; C) HGF; D) and HGF with constant volatility. Both learning rate in latent space, αt, and implied learning rate in the observation space, αto, are plotted. Unlike the former, αto is increased following switches even when volatility is held fixed. However, this behavior does not depend on top-down inferences about volatility, and therefore increases following switches even when volatility is held fixed. See S2 Appendix for a formal proof, demonstrating that αto is contaminated by the absolute value of prediction errors regardless of volatility. Parameters of both models were set at 0.5 except ω of the HGF, which was set at –3. For fixing volatility in VKF and HGF, we assumed λ = 0 and κ = 0, respectively. (PDF) [file pcbi.1007963.s004.pdf]
